# Pharmacological properties and therapeutic potential of berberine: a comprehensive review

**DOI:** 10.3389/fphar.2025.1604071

**Published:** 2025-08-14

**Authors:** Ke-Qin Fan, Liangming Zhang, Fangyu Song, Yue-Hui Zhang, Tong Chen, Xiang Cheng, Ning Su, Yan Zou, Ting Yu, Futing Tan, Wenhao Xu, Zijun Yan

**Affiliations:** ^1^ School of Pharmaceutical Sciences and Yunnan Key Laboratory of Pharmacology for Natural Products, Kunming Medical University, Yunnan, Kunming, China; ^2^ School of Pharmacy, Dali University, Dali, Yunnan, China; ^3^ Department of Pharmacy, Panzhihua Central Hospital, Sichuan, Panzhihua, China

**Keywords:** berberine, pharmacological properties, pharmacodynamics, therapeutic potential, application prospects

## Abstract

In recent decades, the pharmacological properties of botanical drugs have been investigated with increasing depth, offering novel insights into their potential for enhancing healthcare. Berberine (BBR) is an alkaloid extracted from the roots, rhizomes and stem tubers of plants such as *Coptis chinensi*s, *Phellodendron amurense*, *Radix berberidis*, and several other plants, which is used not only as an anti-inflammatory and antibacterial agent, but also for the treatment of cancer and chronic diseases. BBR has demonstrated remarkable therapeutic efficacy in the management of disorders affecting the nervous, cardiovascular, and endocrine systems, characterized by its high safety profile and minimal adverse effects. Despite the substantial progress made in understanding BBR’s pharmacodynamics, its precise mechanisms of action remain incompletely elucidated and warrant further systematic investigation. This study provides an extensive review of the latest pharmacological findings related to berberine and its therapeutic advancements, offering strong evidence for future research and clinical implementation.

## 1 Introduction

In recent years, the pharmacological properties of botanical drugs have been explored and uncovered with increasing depth, opening new avenues for the advancement of healthcare systems. Botanical drugs have long been esteemed for their remarkable efficacy in preventing and treating diseases, positioning them as a crucial pillar of human health (Raskin et al., 2002). According to the World Health Organization, herbal remedies continue to serve as the primary source of healthcare for more than half of the global population, with particular significance in developing nations (Bodeker, 1996; Kim et al., 2020; Mei, 2024). The prominence of botanical drug is largely attributable to its high cultural acceptability, strong compatibility with the human body, and relatively low incidence of adverse effects (Yuan et al., 2023). However, the discovery and processing of plant-based medicinal products also face numerous challenges, including access restrictions, difficulties in material identification, and the conservation of wild species (Ge W. et al., 2024). Despite these hurdles, natural products in certain contexts continue to enjoy broad popularity worldwide due to their perceived ease of use, affordability, renewable resources, and relatively low toxicity in well-studied compounds (Newman and Cragg, 2020).

Among the myriad of natural phytochemicals, berberine (BBR) has garnered significant attention from scholars both domestically and internationally. As an isoquinoline alkaloid isolated from the traditional Chinese medicinal herbs *Coptis chinensis* and Phellodendron amurense, BBR is renowned for its heat-clearing, damp-drying, and detoxifying properties. It has been employed in Chinese medical practice for thousands of years in the treatment of various inflammatory diseases (Yu et al., 2017). Studies have shown that BBR has demonstrated exceptional antimicrobial efficacy in the treatment of intestinal infections, conjunctivitis, and suppurative otitis media. Additionally, it has shown significant therapeutic benefits in the management of chronic conditions such as type 2 diabetes, hyperlipidemia, and hypertension (Riu et al., 2020). Some natural antioxidant agents and their biological properties with anti-oxidative stress potentials in some tissues related to health (Gogebakan et al., 2012; Selamoglu Talas et al., 2008; Adnan et al., 2021; Das et al., 2024), BBR also has a certain antioxidant capacity (Salehi et al., 2019). Notably, BBR is characterized by a favorable safety profile, with only mild and infrequent gastrointestinal discomfort reported as side effects, rendering it an optimal treatment option for patients with limited financial resources (Nie et al., 2024). Despite significant progress in BBR research, a comprehensive and systematic elucidation of its exact pharmacological mechanisms and its role in disease treatment remains lacking. Current studies tend to focus on a specific pharmacological activity of BBR for particular diseases, and there is a lack of a comprehensive article that integrates the latest research findings to fully reveal its pharmacological mechanisms in disease treatment.

**FIGURE 1:**
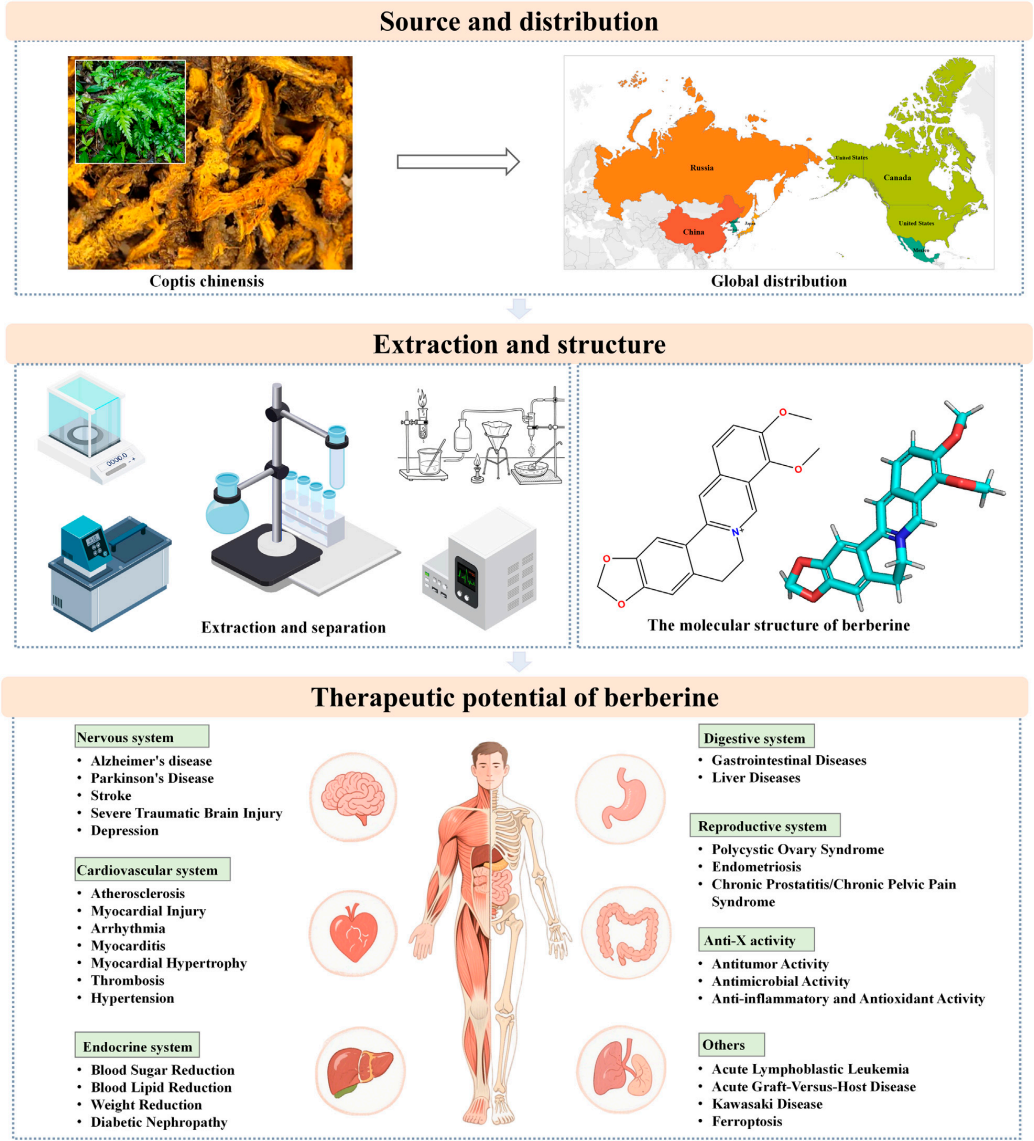
Technological road-map.

This study aims to provide an overview of the sources, chemical composition, and bioactive constituents of BBR, as well as its extraction and isolation methods. It comprehensively discusses the mechanisms underlying BBR’s protective effects in a range of diseases affecting the nervous, cardiovascular, endocrine, digestive, and reproductive systems (Figure 1). Additionally, the study summarizes BBR’s bioactivity in anti-tumor, anti-inflammatory, antimicrobial, and antioxidant activities, with the goal of providing robust evidence for its further research and clinical application. Through this review, a more comprehensive understanding of BBR’s clinical potential and pharmacological mechanisms across various diseases is presented, highlighting key areas such as neuroprotection, cardiovascular protection, glycemic and lipid-lowering effects, and anti-cancer properties.

## 2 Literature retrieval and screening methods

### 2.1 Language

The pharmacological mechanisms of action section of this review includes only studies originally written in English; other sections such as source, extraction and separation include Chinese studies.

### 2.2 Databases

PubMed databases were searched. The mesh terms used were berberine or umbellatine and pharmacological mechanism of action or mechanisms of pharmacological action or pharmacology or mechanism of action or mode of action or pharmacologic action or molecular mechanisms of pharmacological action. The mesh terms enabled the search and identification of *in vivo* and *in vitro* studies that were related to the objective of this review. Chinese studies were obtained through CNKI database.

### 2.3 Study selection

In this review, due to the lack of clinical studies of BBR, both *in vitro* and *in vivo* studies were considered in the final analysis. Only full texts were considered. Studies on BBR derivatives, novel delivery systems, and network pharmacological analysis were excluded.

### 2.4 Time frame

The studies included in the pharmacological mechanism of action section were mainly published from 2017 to 2024.

## 3 Source

BBR is an alkaloid compound extracted from the roots, rhizomes, and stem bulbs of plants belonging to the *Berberidaceae*, *Ranunculaceae*, and *Papaveraceae* families (such as *Coptis chinensis*, *Phellodendron amurense*, *Radix berberidis*, and several other plants). In addition, studies have shown that BBR can also be extracted from plants of the *Zanthoxylum monophyllum* (Stermitz and Sharifi, 1977). It is the principal alkaloid component in *C. chinensis*. In addition to being extracted from natural sources, BBR can also be synthetically produced through modern manufacturing processes (An et al., 2022). Studies have shown that there are 11 species and one subspecies of *Coptis* globally. Among these, six species are found in China, with another 6 to 8 species in Japan and the Russian Far East, and 4 species in North America (Chen, 2022).

## 4 The physical and chemical properties of berberine

BBR is a yellow, needle-like crystalline compound that precipitates in ether. It is odorless, with an intensely bitter taste, and has a melting point of 145°C. It is readily soluble in hot water, sparingly soluble in cold water and ethanol, and insoluble in benzene, chloroform, and acetone. Classified as a quaternary ammonium compound and an isoquinoline alkaloid, its clinical applications are primarily in the form of hydrochloride and sulfate salts (Si et al., 2019). The chemical name of BBR is 5,6-dihydro-9,10- dimethoxybenzo[g]-1,3-benzodioxolo[5,6-á]quinolizine, with a molecular structure of C20H18NO4 and a molecular weight of 336.39 g/mol. Pharmacokinetic studies indicate that BBR exhibits a very low plasma drug concentration following oral administration, with extensive distribution throughout the body, rapid metabolism, and slow elimination (Hui and Mao, 2011). After oral intake, BBR is rapidly converted into phase I metabolites, which are subsequently conjugated with glucuronic acid or sulfate to form phase II metabolites. These metabolites are ultimately excreted through urine and bile. The main metabolic pathways of BBR include demethylation and glucuronidation, but the bioavailability of BBR itself is low (Wang K. et al., 2017).

## 5 Extraction of berberine

The extraction methods commonly used in BBR include Solvent Extraction, Ultrasound-assisted method, Microwave-assisted method, Ultrasonic-microwave synergistic method, Low eutectic solvent method and Accelerated solvent extraction (Table 1).

**TABLE 1:** Extraction of berberine.

Methodology	Processes/Optimal processes	References
Solvent Extraction - Aqueous extraction	Coptidis Rhizoma pieces were immersed in 8 volumes of 1.5% sulfuric acid solution and decocted three times; each decoction lasted 40 min	Yao et al. (2012)
Ultrasound-assisted method	The optimal conditions were determined as an extraction time of 35 min, a moisture content of 26%, a liquid-to-material ratio of 33:1	Jie et al. (2024)
Microwave-assisted method	Irradiation time: 4 min, sulfuric acid concentration: 0.06 mol/L, material-to-liquid ratio: 1:80	Erjin et al. (2013)
Ultrasonic-microwave synergistic method	The extraction solvent was 0.05 mol/L H_2_SO_4_, the material-to-liquid ratio was 15 g/mL, the ultrasonic extraction time was 10 min, the microwave extraction time was 3 min, and the microwave power was set to 600 W	Xin et al. (2023)
Low eutectic solvent method	Betaine to lactic acid ratio is 1:3, with a low eutectic solvent water content of 30%. The material-liquid ratio is 1:20 and the extraction time is 30 min	Ming et al. (2023)
Accelerated solvent extraction	The extraction solvent was set as 80% (v/v) aqueous ethanol containing 0.5% (w/v) HCl, with extraction conducted at 130°C for a static extraction duration of 10 min in a single extraction cycle	Heng et al. (2008)

## 6 Separation of berberine

The separation methods commonly used in BBR include Macroporous adsorption resin method, High-speed countercurrent chromatography, Ion-exchange fiber method, Column chromatography and Non-capillary electrophoretic separation (Table 2).

**TABLE 2:** Separation of berberine.

Methodology	Processes/Optimal processes	References
Macroporous adsorption resin method	The 80% (v/v) aqueous ethanol containing 0.3% (w/v) NaCl completely desorbed alkaloids from Coptidis Rhizoma-loaded PNaA resin under static conditions, yielding 100% desorption efficiency	Bei et al. (2020)
High-speed countercurrent chromatography	The crude extract of Coptidis Rhizoma obtained by ethanol extraction underwent a four-step purification sequence via high-speed countercurrent chromatography, yielding an extraction rate of 68.54%	Mao et al. (2022)
Ion-exchange fiber method	The ion-exchange fiber demonstrated adsorption and desorption efficiencies of 97.93% and 83.17%, respectively, for BBR.	Sun et al. (2009)
Column chromatography	The purity of berberine hydrochloride purified by this method is above 97%	Ji (2006)
Non-capillary electrophoretic separation	The optimal electrophoresis buffer was a 50 mM ammonium acetate methanol solution containing 0.5% (v/v) acetic acid and 10% (v/v) acetonitrile, applied at a voltage of 18 kV. Analytes were detected by UV light at 214 nm	Gao et al. (2005)

## 7 Pharmacological mechanisms and disease treatment of berberine

### 7.1 Neuroprotective effects

**FIGURE 2:**
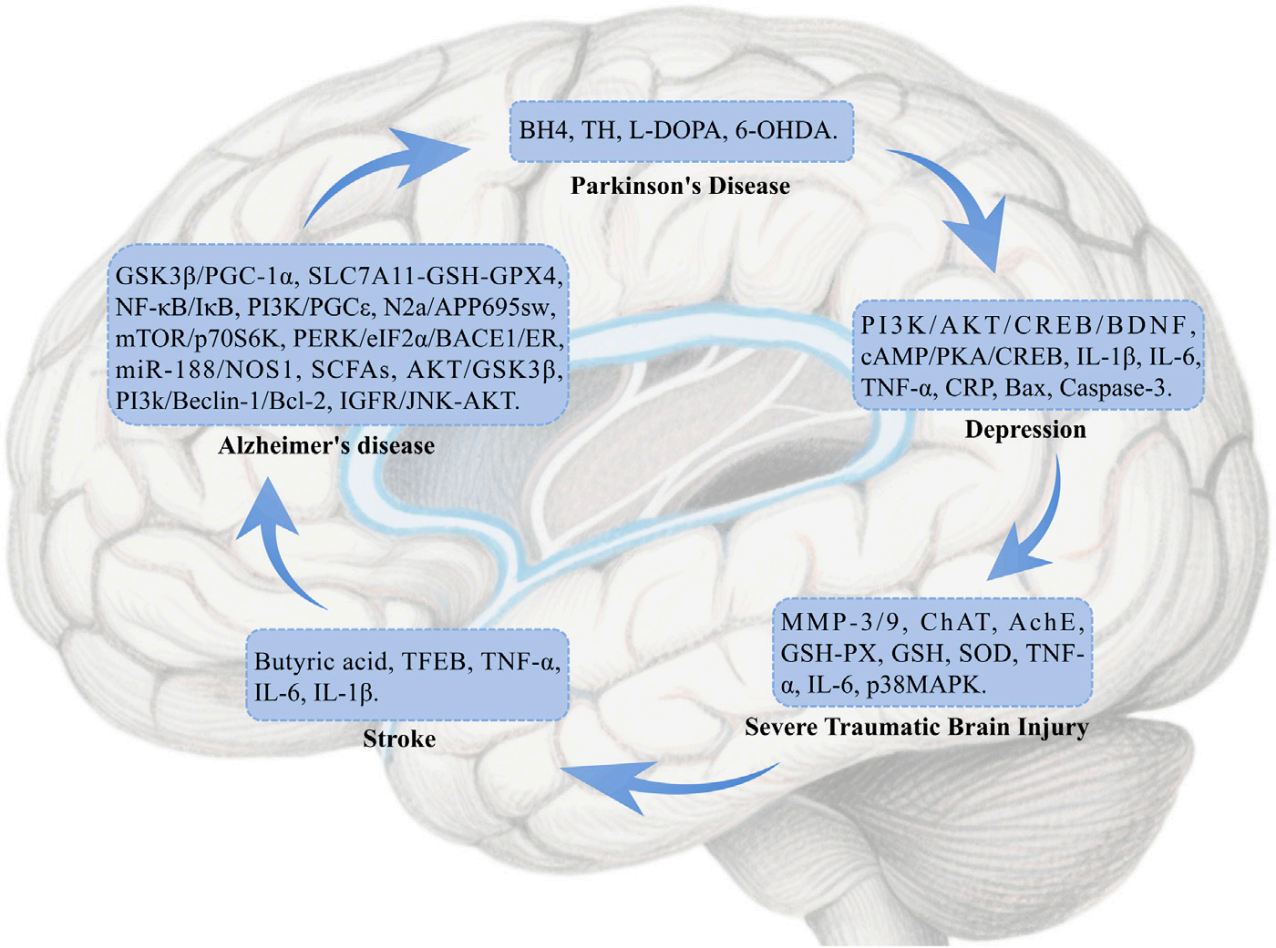
The mechanism of berberine’s action on the Nervous system.

Neurological diseases are disorders that affect the brain, spinal cord, nerves, and muscles, encompassing conditions such as cerebrovascular diseases, neuroimmune diseases, infections, and others that can lead to motor, sensory, and cognitive impairments (Shayganfard, 2023). These diseases are caused by various factors, including genetics, infections, and trauma. This section explores the pharmacological mechanisms of BBR in treating Alzheimer’s Disease (AD), Parkinson’s Disease (PD), stroke, traumatic brain injury (TBI), depression, and other related conditions (Figure 2).

#### 7.1.1 Alzheimer’s disease

AD is a complex neurodegenerative disorder that induces a variety of cellular changes, including cholinergic system dysfunction, aggregation of β-amyloid proteins, hyperphosphorylation of Tau, dysregulation of metal homeostasis, and neuroinflammation. BBR enhances antioxidant activity through multiple pathways and targets, reduces inflammation-related biomarkers, promotes the formation of microvessels in the brain, and facilitates the creation of structurally intact and functionally competent new blood vessels. These actions help restore cerebral blood flow, regulate the gut-brain axis via short-chain fatty acids (SCFAs), and ultimately ameliorate AD-like cognitive impairments and spatial memory dysfunction (Patil et al., 2020) (Table 3).

**TABLE 3:** The mechanism of berberine’s action on Alzheimer’s disease.

Promote/increase	Suppression/reduction	Conditioning pathway	Implication	References
PGC-1α, Aβ-degrading enzymes and Neprilysin	GSK3β, IL-1β, Aβ plaques and deposition, Tau hyperphosphorylation, caspase-3, the activity of BACE1 and γ-secretase	GSK3β/PGC-1α, mTOR/p70S6K	Improvement of AD-like cognitive impairment and reduction of neuronal damage	Yang and Wang (2022), Wang et al. (2021a)
Nrf 2	Fe^2+^ level	SLC7A11-GSH-GPX4	Inhibition of iron death	Li et al. (2023a)
AKT and ERK phosphorylation levels	Formation of pathogenic NFTs		Protecting hippocampal neurons	Wei et al. (2023)
	IκB degradation	NF-κB/IκB	Inhibition of NF-κB binding to target DNA to block NF-κB transcriptional activity	Fang et al. (2020)
LKB1/AMPK pathway, Aβ clearance, brain platelet endothelial cell adhesion molecule-1, vascular endothelial growth factor, CD31, VEGF, N-cadherin, Ang-1	NFT formation and Aβ breakdown, NF-κB, Tau hyperphosphorylation, APP, BACE1	PERK/eIF2α/BACE1/ER, miR-188/NOS1, N2a/APP695sw	Promotes the restoration of cerebral blood flow	Huang et al. (2023), Ye et al. (2021)
Pi3K, GLUT3, PKCε	p-IRS, APP, GSK3βY216, Generation of the oligomer Aβ42	PI3K/PGCε	Lengthening of neuronal axons, amelioration of neuronal axonal damage	Wu et al. (2021)
Autophagic flux, Tau clearance	Tau hyperphosphorylation	AKT/GSK3β, PI3k/Beclin-1/Bcl-2	Mitigating cognitive decline	Chen et al. (2020a)
GPx, SOD, catalase, GSH	AChE, MDA, protein carbonyls, cysteine 3 activity and DNA fragmentation in hippocampal activity, NF-κB, TLR4, TNFα, IL-6, oxidative nitrosative stress, recovery of AChE, MAPK and sirtuin 1		Boosts antioxidant capacity, decreases inflammation, improves cognitive deficits induced by LPS, and provides neuroprotective effects	Sadraie et al. (2019)
Acetic acid, ILA		SCFAs, Histidine and phenylalanine metabolic pathways	Acting on the gut-brain axis to treat AD.	Xie et al. (2023)
IGFR	JNK, AKT	IGFR/JNK-AKT	Promotion of nerve regeneration	Zhang et al. (2018a)

#### 7.1.2 Parkinson’s disease

BBR stimulates the biosynthesis of tetrahydrobiopterin in the gut microbiota, increases dopamine and L-DOPA concentrations in both the blood and brain, enhances tyrosine hydroxylase activity to produce L-DOPA, and regulates the biosynthesis of phenylalanine, tyrosine, dopamine, and other intermediates, thus improving brain function and overall motor abilities in animals (Wang et al., 2021b). It also significantly depletes the number of tyrosine hydroxylase-positive cells in the substantia nigra and reduces dopamine and norepinephrine levels in the striatum, affecting PD (Kwon et al., 2010). BBR protects against 6-OHDA-induced cell death, attenuates MPTP-induced Parkinson’s disease-like behaviours and dopaminergic neuron loss in zebrafish by targeting cerebral mitochondria via mitophagy regulation (Wang L. et al., 2021).

#### 7.1.3 Stroke

BBR may significantly increase the abundance of beneficial bacteria producing butyrate by modulating the gut microbiota, thereby enhancing butyrate levels. This action suppresses the activation of microglia and astrocytes in the brain of model mice and inhibits the production of pro-inflammatory cytokines (IL-6, IL-1β, TNF-α), ultimately improving stroke outcomes (Duan et al., 2023). Additionally, BBR promotes the nuclear translocation of TFEB in neurons, increases autophagic flux, and enhances both autophagic activity and lysosomal function to mitigate ischemic injury and protect against ischemic stroke (Liu Y. et al., 2024).

#### 7.1.4 Severe traumatic brain injury

BBR reduces cerebral edema and inhibits the expression of MMP-3/9 proteins, promoting ChAT activity and inhibiting AchE activity in mice with severe TBI. This results in a significant increase in the activities of GSH-PX, GSH, and SOD, exerting antioxidant effects. BBR also diminishes the levels of inflammatory cytokines TNF-α and IL-6 and generates neuroprotective effects by inducing the expression of SIRT1 and inhibiting p38 MAPK expression. These actions help restore learning and memory abilities in severe TBI mice (Wang and Zhang, 2018).

#### 7.1.5 Depression

BBR improves depressive-like symptoms in chronic restraint stress mice by upregulating the phosphorylation and mRNA expression of PI3K and AKT, which subsequently increases the mRNA and protein expression/phosphorylation of CREB. This effect likely occurs through the PI3K/AKT/CREB/BDNF signaling pathway. Additionally, BBR regulates the mRNA expression levels of IL-1β, IL-6, TNF-α, CRP, Bax, and Caspase-3, suppresses inflammation and cell apoptosis, and alleviates cell damage induced by corticosterone (Tang et al., 2024a). Further studies indicate that BBR improves diabetes and depression by activating the cAMP/PKA/CREB signaling pathway (Tang et al., 2024b).

### 7.2 Cardiovascular protective effects

**FIGURE 3:**
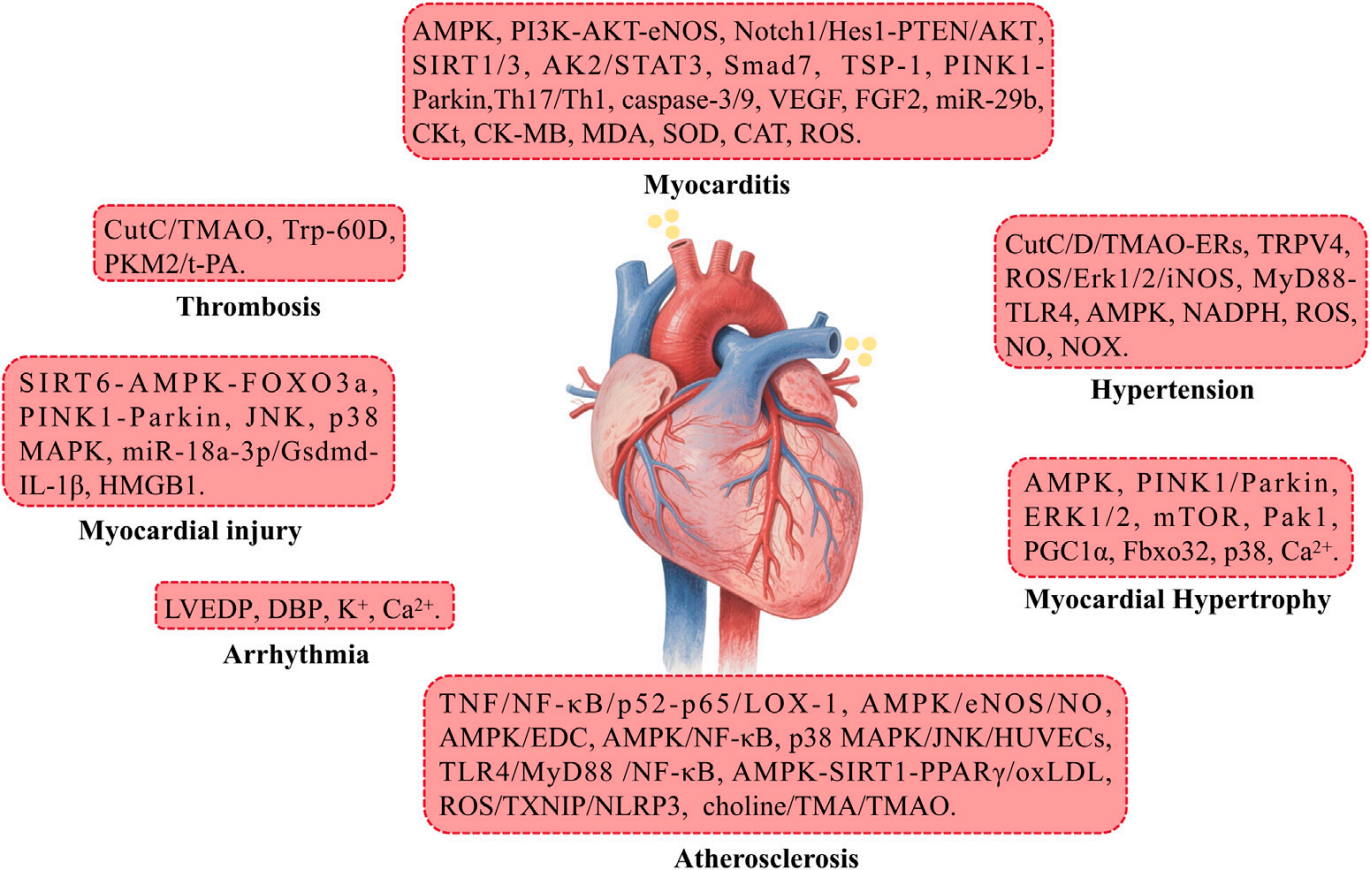
The mechanism of berberine’s action on the Cardiovascular system.

Cardiovascular diseases refer to conditions that affect the structure and function of the heart and blood vessels, including coronary heart disease, hypertension, myocardial infarction, arrhythmias, and others. These diseases often lead to circulatory disorders, manifesting symptoms such as chest pain, palpitations, and shortness of breath. In severe cases, they may be life-threatening and represent a major global health issue (Feng et al., 2019). This section primarily discusses the therapeutic approaches of BBR in treating cardiovascular diseases such as atherosclerosis (AS), myocardial injury, myocardial hypertrophy, and hypertension (Figure 3).

#### 7.2.1 Atherosclerosis

BBR plays a role in inhibiting atherosclerosis by improving endothelial dysfunction, suppressing smooth muscle cell proliferation and migration, reducing monocyte adhesion, macrophage inflammation, cholesterol accumulation, foam cell formation, and platelet aggregation. Additionally, BBR prevents ischemia/reperfusion injury by increasing positive inotropic activity, enhancing the phosphorylation of Bad, reducing the production of pro-inflammatory mediators (IL-6, IL-1β, and TNFα), alleviating oxidative stress, lowering blood pressure, and providing protection against endoplasmic reticulum (ER) stress (Table 4) (Feng et al., 2019).

**TABLE 4:** The mechanism of berberine’s action on atherosclerosis.

Promote/increase	Suppression/reduction	Conditioning pathway	Implication	References
Expression of the anti-apoptotic protein myeloid cell leukemia-1	visceral adiponectin levels, lipid levels, liver lipid accumulation	TNF/NF-κB/p52-p65/LOX-1, AMPK/NF-κB, p38MAPK/JNK/HUVECs	Amelioration of endothelial mesenchymal thickening and endothelial damage	Kamaruddin et al. (2022)
	NOX2, NOX4, COX-2, ROS, ERs	AMPK/eNOS/NO, AMPK/EDC	Improves vasoconstriction	Amssayef and Eddouks (2023a)
LOX-1, AMPK/mTOR	CD68, MMP9, EMMPRIN, TNF-α, SR-BI	TLR4/MyD88/NF-κB, AMPK-SIRT1-PPARγ/oxLDL	Inhibits monocyte migration and foam cell formation during migration	Rui et al. (2021), Cai et al. (2021)
KLF16, PPARα			Reducing diabetes AS	Man et al. (2022)
NLRP3, ZO-1, VEC, TXNIP	caspase 1, IL-1β, HMGB1, Ca^2+^ responds to ATP inward flow	ROS/TXNIP/NLRP3	Improvement of inflammatory vascular injury	Man et al. (2022), Dai et al. (2022)
	TMAO, cutC, cntA	choline/TMA/TMAO	Reduce the development of AS	Li et al. (2021a)

#### 7.2.2 Myocardial injury

BBR modulates several targets and signaling pathways to alleviate myocardial injury, heart failure, and cardiomyopathy. Specifically, BBR induces the expression of miR-181c-5p and miR-340-5p while inhibiting HMGB1 expression, thereby promoting the expression of miRNAs targeting HMGB1, effectively reducing cancer-related myocardial injury (Goto et al., 2024). BBR also activates the SIRT6-AMPK-FOXO3a signaling pathway, enhances PINK1-Parkin-mediated mitochondrial autophagy to reduce oxidative stress, and preserves mitochondrial function. In a dose-dependent manner (Zhou et al., 2024), BBR inhibits CIH-induced myocardial remodeling, heart failure, and myocardial injury. Moreover, BBR upregulates miR-18a-3p to suppress miR-18a-3p-mediated Gsdmd activation, IL-1β secretion, and alleviates diabetic cardiomyopathy (Yang et al., 2023). Additionally, BBR suppresses CVB3r replication by inhibiting the p38 MAPK and JNK pathways, thus reducing cardiac damage (Hashemzaei et al., 2017).

#### 7.2.3 Arrhythmia

BBR increases cardiac output, reduces LVEDP and DBP, and prevents heart failure. It also prevents arrhythmias by decreasing the frequency of ventricular premature beats and tachycardia. Furthermore, BBR inhibits K^+^ and Ca^2+^ current activation, thus prolonging the effective refractory period of the atria and the action potential duration of atrial myocytes (Cai et al., 2021).

#### 7.2.4 Myocarditis

BBR activates several signaling pathways, including AMPK, PI3K-AKT-eNOS, Notch1/Hes1-PTEN/AKT, AK2/STAT3, SIRT1, and Smad7, finely regulates the PINK1-Parkin pathways, and effectively reduces cell apoptosis and oxidative stress. It inhibits myocardial inflammation, promotes mitochondrial autophagy, and myocardial cell proliferation, significantly reducing I/R damage (Abudureyimu et al., 2020). Moreover, BBR inhibits the expression of caspase-3, upregulates VEGF, FGF2, and TSP-1 expression, and promotes ischemia-induced angiogenesis through the upregulation of miR-29b, reducing infarct area and improving heart function (Zhu et al., 2017). Furthermore, BBR suppresses Th17/Th1 cell differentiation, intracellular Ca^2+^ levels, caspase-9 and -3 activation, decreases CKt, CK-MB, and MDA levels, while increasing SOD, CAT, SIRT3 levels, upregulating SIRT1, and downregulating p66shc expression. This synergistically improves left ventricular function, mitigates cardiac toxicity, suppresses ROS production, apoptosis, mitochondrial damage, and cardiac fibrosis, comprehensively improving heart function (Hashemzaei et al., 2017).

#### 7.2.5 Myocardial hypertrophy

BBR activates the AMPK signaling pathway to inhibit mitochondrial fission, upregulates PGC1α to stimulate mitochondrial biogenesis, restores autophagic flux, and interferes with the prevention of high glucose-induced myocardial hypertrophy. Additionally, BBR may regulate the mTOR pathway to suppress the phosphorylation of ERK1/2 and p38, increase autophagy, alleviate ER stress, and activate the Pak1 pathway to inhibit Fbxo32 upregulation, lower myocardial cell Ca^2+^ concentration, reduce left ventricular end-diastolic pressure, upregulate PINK1/Parkin-mediated mitochondrial autophagy, and thus inhibit myocardial cell apoptosis and mitochondrial damage, improving heart failure and myocardial hypertrophy (Abudureyimu et al., 2020).

#### 7.2.6 Thrombosis

BBR reduces thrombus formation by inhibiting CutC enzyme activity and decreasing TMAO production (Qu et al., 2024). It also improves thrombus formation by suppressing the expression of PKM2, thereby influencing the expression of tissue t-PA in the fibrinolytic system (Sun Z. et al., 2024).

#### 7.2.7 Hypertension

BBR inhibits the biosynthesis of TMAO precursors in the gut microbiota by binding to and inhibiting the activity of CutC/D enzymes, downregulates the TMAO-endoplasmic reticulum stress pathway, and alleviates endothelial cell dysfunction, thereby improving vascular function. Simultaneously, BBR inhibits TRPV4 and MyD88-TLR4 signaling pathways, relaxes vascular smooth muscle, protects endothelial cells from damage, increases NO expression to promote vasodilation, and activates AMPK to inhibit endoplasmic reticulum stress in endothelial cells, safeguarding vascular function. BBR also prevents NADPH oxidase expression and ROS production, increases NO bioavailability, forms stable free radicals with ROS-derived NADPH oxidase, and prevents NOX subunit assembly, thus improving hypertension (Cai et al., 2021; Qu et al., 2024; Yousefian et al., 2019; Wang Z. et al., 2024) Furthermore, BBR suppresses the ROS/Erk1/2/iNOS pathway to alleviate hypertension and sympathetic nervous excitation in double-kidney, one-kidney hypertension rat models (Tian H. et al., 2019).

### 7.3 Protective effects on the endocrine system

**FIGURE 4:**
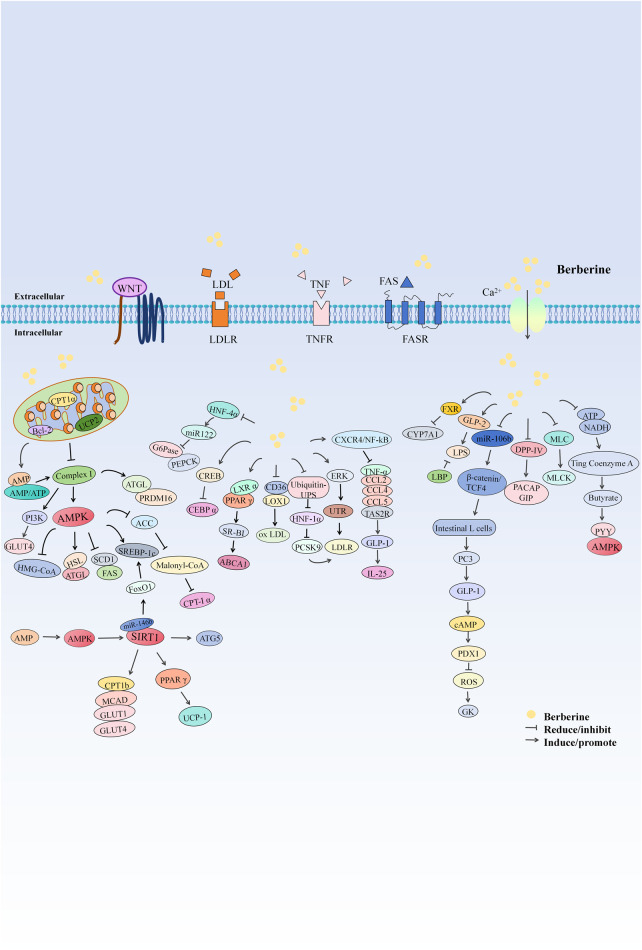
The mechanism of berberine’s action on the Endocrine system.

Endocrine disorders involve diseases that affect endocrine glands or tissues, leading to abnormal hormone secretion and triggering a range of symptoms. These disorders include diabetes, thyroid diseases, and pituitary tumors, often manifesting as metabolic disorders, growth abnormalities, and significantly impacting patient health and quality of life. This section discusses the activities of BBR in reducing blood sugar (Shrivastava et al., 2023), lowering blood pressure (Alruhaimi et al., 2024), and decreasing weight (Wang H. et al., 2024), among other effects (Figure 4).

#### 7.3.1 Blood sugar reduction

Numerous studies have confirmed the hypoglycemic activity of BBR and identified various mechanisms through which it lowers blood sugar. These include promoting insulin secretion, alleviating insulin resistance, inhibiting gluconeogenesis, enhancing glucose uptake, improving inflammation, and regulating gut microbiota disturbances. BBR also improves gut-brain communication, relieves intestinal epithelial barrier dysfunction, and enhances intestinal permeability, thereby aiding in the amelioration of obesity and insulin resistance-related metabolic abnormalities (Table 5).

**TABLE 5:** The mechanism of berberine in lowering blood sugar.

Promote/increase	Suppression/reduction	Conditioning pathway	Implication	References
GLP-1, GLP-2		JNK/NF-ĸB	Protects pancreatic β cells	Amssayef and Eddouks (2023b)
AMPK, IGF-1, Min6, Methylation of miR-106b, GLP-1, GnRHGK.	miR-106b, DPP-Ⅳ	β-catenin/TCF4, cAMP/PDX1/ROS/GLP-1, DPP-IV/PACAP-GIP, PLC/STC-1/TAS2Rs, PKC/NCI-H71/GCGmRNA/PC3	Promotes insulin secretion	Zhou et al. (2019), Wang et al. (2021d), Zhang et al. (2014), Wang et al. (2016)
PKC, InsR, PGC-1α, SIRT1, SIRT3, GLUT4, pAKT/AKT, FoxO1, PDX1, GLUT2, GPR40, AMP/ATP, Nrf2, AMPK phosphorylation, LXRs, PPARs, Cyp7a, PI3K, GLUT2, ADSL, Ntcp, FXR.	PPARγ, FAT/CD36, miR-27a, LPS/TLR4/TNF-α, HPA, CORT, AMPD1, TGF-B, SREBPs, TLR4, p-JNK, BCAA.	miR-146b/SIRT1, LBP/LPS, GLP2, AKT/GLUT4, AMPK/SIRT1/PPARγ/UCP-1, IKK/NF-κB, JNK, IRS-1/AKT.	Improves insulin resistance	Gu et al. (2010), Kong et al. (2009), Su et al. (2023), Sui et al. (2021), Du et al. (2024), Liu et al. (2018), Mi et al. (2019), Koperska et al. (2022), Cui et al. (2018), Cheng et al. (2023), Xu et al. (2021a), Yue et al. (2019), Gu et al. (2012)
p-AMPKα1, p-AMPKα, AMPK.	α-glucosidase	Complex I/AMP/AMPK, C2C12/AKT1/GLUT1, RBP4, AMPK/LCA-MCAD-CPT1b/SIRT1/GLUT1-GLUT4	Enhances glucose uptake	McCarty and DiNicolantonio (2021), Hou et al. (2018), Gomes et al. (2012), Cok et al. (2011)
FXR, LKB1, AMPK, p-AMPK, HNF-4α mRNA, TLR4	PEPCK, G6Pase, FBPase, SIRT3, ATP/ADP, ATP/AMP.	LKB1-AMPK-TORC2, AKT1/MAPK/NO/cGMP/PKG, PDE/cAMP/CREB, SIRT3/MPC1/Mitochondrial pyruvate supply, FOXO1/PEPCK -G6Pase	Inhibits gluconeogenesis	Gupta et al. (2024), Ilyas et al. (2020), Xia et al. (2011), Jiang et al. (2015), Wei et al. (2016), Zhang et al. (2018b), Li et al. (2018), Lu et al. (2023)
AMPK, Nrf2, GLP-1, Ghrelin-A, GLP-2 , ZO-1mRNA , Abundance of beneficial gut bacteria, B/F ratio, balance of gut microbiota	CRP, IL-6, TNF-α, IL-1b, PKB-NF-κB, ATP, NADH, SCFA, LPS, TLR-4, NF-κB, TNF-α	ATP-NADH/Butyryl-CoA/butyrate/PYY/AMPK, MTORC, TNF-α-IFN-γ/Caco-2/ZO-1-occludin-claudin-MLC-MLCK, LPS/TLR4/TNF-α/BSH.	Alleviates inflammation, modulates gut microbiota composition	Zhao et al. (2021), Yan et al. (2022), Sun et al. (2017a), Wang et al. (2021e), Xu et al. (2017), Han et al. (2011)

#### 7.3.2 Blood lipid reduction

Elevated blood lipid levels are a common feature of lipid metabolism disorders such as obesity, hyperlipidemia, and atherosclerosis. BBR can reduce blood lipid levels by inhibiting the progression of hyperlipidemia, reducing adipocyte differentiation, decreasing triglyceride (TG) accumulation, inhibiting the synthesis of total intracellular triglycerides; improving cholesterol esters (CE), inhibiting cholesterol synthesis, lowering cellular cholesterol levels; reducing circulating low-density lipoprotein and cholesterol levels, promoting bile acid uptake, and enhancing intestinal barrier function. These actions significantly lower blood lipid levels and alleviate dyslipidemia (Table 6).

**TABLE 6:** The mechanism of berberine in lowering blood lipids.

Promote/increase	Suppression/reduction	Conditioning pathway	Implication	References
MTTP, MAPK, VLDL	FAS, PPARγ, ADD1/SREBP1c, 11β-HSD1	AMPK-SREBP-1c/SCD1, 3T3L/complexI/AMPK/ATGL-HSL	Reduces triglycerides	Lee et al. (2006), Chen et al. (2021), Li et al. (2020), Yang et al. (2022)
HMGCR, Gut genus Blautia, conjugated bile acids, PPAR γ, LXR α, SR-BI, ABCA1	acyl-CoA, ACAT2, AEBP1, Gut genus Alistipes, BSH	AMPK/HMG-CoA, UPS/HNF-1α/PCSK9/LDLR, Nrf2/HO-1, PKCδ/ABCA1, FXR.	Lowers cholesterol	Och et al. (2022), Wang and Zidichouski (2018), Ge et al. (2024b), Dong et al. (2015), Pirillo and Catapano (2015), Liang and Wang (2018), Wang et al. (2024c), Cai et al. (2023), Nourizadeh et al. (2022), Wang et al. (2014)
LDLR		JNK-ERK/UTR-PCSK9/LDLR	Lowers LDL	Amssayef and Eddouks (2023b), Sun et al. (2021), Patti et al. (2019)
FXR, TCA, Bacs mRNA, Baat mRNA, NTCP	*Clostridium* cluster XIVa and IV and BSH activity, Ntcp and Oatp1	FXR, STAT5	Inhibits bile acids	Tian et al. (2019b), Bu et al. (2017)
MCADD, ABCA1, ABCA1/G1, LDLR, APOEmRNA	PPAR γ, CEBP α, CREB, HMGR, MDA, SOD, GSH-px, LOX-1	AMPK/ACC/Malonyl-CoA/CPT-Iα, SREBP1-ChREBP/FAS, LXRα-ABCA1/oxLDL, AMPK/PI3K/GLUT4, AMPK/ACC-FAS-GPAT	Alleviates dyslipidemia, blocks fat formation	Alruhaimi et al. (2024), Lee et al. (2006), Chen et al. (2021), Yang et al. (2012), Zhou et al. (2016a), Tang et al. (2006), Xu et al. (2019)
SCFAs-producing bacteria, Na^+^/H^+^, TGR5, GLP, CYP7A1, CYP27A1, bile acids-decomposing bacteria, phyla Firmicutes and Actinobacteria, GLP-1, GLP-2, L cells, NR-producing bacteria, Bacteria degrading mucins	CD14, IL-1, IL-6, TNF-α, Planomicrobium, BSH	NASH, HMGCT, SREBP2, Microbiome-gut-brain axis	Improves intestinal barrier function, enhances the lipophilicity and efficacy of berberine, significantly reduces blood lipids	Kong et al. (2022), Cao et al. (2016), Wang et al. (2022a)

#### 7.3.3 Weight reduction

In addition to its hypoglycemic and lipid-lowering effects, studies have found that BBR can effectively improve obesity and reduce body weight by reducing adipocyte formation, decreasing fat production, increasing fat breakdown, and promoting adipose tissue remodeling (Table 7).

**TABLE 7:** The mechanism by which berberine reduces weight.

Promote/increase	Suppression/reduction	Conditioning pathway	Implicati on	References
AMPK, PRDM16, ATGL	Mitochondrial respiration, ALT	AMPK-GLUT4/FAO/ATGL, PRDM16/PPARγ/PGC-1α	Inhibits fat formation and promotes total body energy expenditure	Wu et al. (2019)
		SIRT1/ATG5	Enhances autophagy, reduces liver fat storage	Yu et al. (2021a), Sun et al. (2018a)
GATA-2, GATA-3	PPAR γ, CEBP α		Inhibits adipocyte differentiation	Hu and Davies (2010)
ISR, GDF15		BAT-ISR-GDF15	Promotes the development of BAT	Wu et al. (2019)
Levels of PPAR γ deacetylation, UCP-1		AMPK/SIRT1-PPARγ-UCP-1	Promotes adipose tissue remodeling	Xu et al. (2021a)
SREBP-2	LXRs, PPARs, SREBPs, TNFα, CCL2, CCL4, CCL5	ATM-CXCR4/NF-κB-TNFα/CCL2/CCL4/CCL5, CXCR4/NF-κB	Improves obesity and reduces body weight	Li et al. (2011), Noh et al. (2022), Neyrinck et al. (2021)
GLP-1, IL-25	TAS2R	TAS2R-GLP-1, triglyceride levels, and lipid droplet volume	Promotes the proliferation of cluster cells	Sun et al. (2022)
GLP-1, GLP-2, PYY, Lactic acid	ROCK	GLP-1/GLP-2/PYY	Reduces food intake, improves obesity	Zhao et al. (2008)
	SCD1, FABP1, CD36, CPT1A, Gal-3	LEP	Promote fatty acid consumption, β-oxidation, and reduce fat synthesis	Qiu et al. (2022), Wang et al. (2019)

#### 7.3.4 Diabetic nephropathy

BBR lowers the expression and secretion of various inflammatory cytokines in the renal cortex and throughout the body, such as TNF-α, IL-1β, IL-6, and MCP-1. It also inhibits the expression of proteins related to the TLR4/NF-κB pathway, including TLR4, p65, and IKBα. These actions lead to reduced podocyte apoptosis, thickened glomerular basement membrane, enhanced kidney function, and ultimately, alleviated podocyte injury in diabetic nephropathy models (Wu et al., 2024).

### 7.4 Protective effects on the digestive system

**FIGURE 5:**
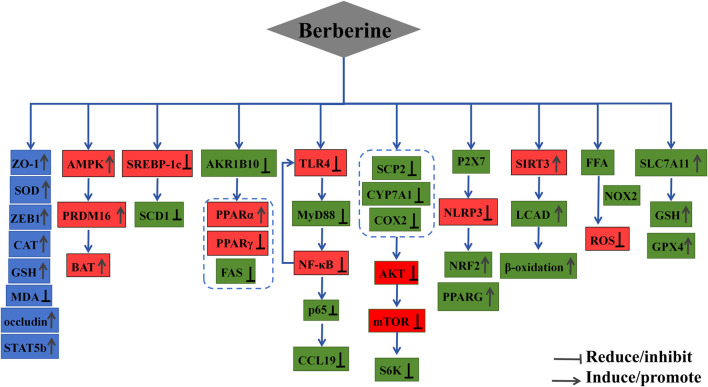
The mechanism of berberine’s action on the Digestive system.

Diseases of the digestive system involve the esophagus, stomach, intestines, liver, and gallbladder, with common ailments including gastritis, gastric ulcers, hepatitis, and cholecystitis. Symptoms include abdominal pain, diarrhea, nausea, and vomiting. These diseases affect nutrient absorption and digestion and may lead to severe complications such as bleeding and perforation (Zou et al., 2017). This paper briefly discusses the treatment of gastrointestinal diseases and hepatitis with BBR (Figure 5).

#### 7.4.1 Gastrointestinal diseases

BBR enhances the expression of ZO-1, ZEB1, occludin, and STAT5b, improving intestinal barrier function (Han et al., 2024). Additionally, BBR treatment significantly increases SOD, CAT, and GSH levels, and reduces MDA levels, potentially protecting against oxidative stress in indomethacin-induced gastric tissue damage (Xu and Yang, 2024).

#### 7.4.2 Liver diseases

BBR reduces inflammation, oxidative reactions, and oxidative stress, enhances aerobic lipid metabolism and mitochondrial function, regulates gut microbiota and bile acid metabolism, alleviates hepatic steatosis, inhibits the progression of non-alcoholic steatohepatitis (NASH) and liver fibrosis, and protects against non-alcoholic fatty liver disease (NAFLD) (Table 8).

**TABLE 8:** The mechanism of berberine’s action on the liver disease.

Promote/increase	Suppression/reduction	Conditioning pathway	Implication	References
AMPK, phosphorylation of SREBP-1c, SRE, SIRT3, p-AMPK, p-ACC, CPT-1A	Expression of SCD1 and other TG synthesis-related genes, gluconeogenesis, endoplasmic reticulum stress	AMPK-SREBP-1c-SCD1, AKR1B10/PPARα-PPARγ/FAS	Reduces hepatic TG synthesis, alleviates hepatic steatosis	Yang et al. (2024), Zhao et al. (2017a), Zhang et al. (2019)
	Activation of ERK1/2 mediated by ER stress, PA/LPS, Angptl2, Foxo1, CCR2, NF-κB		Inhibits inflammatory response in NAFLD hepatocytes	Wang et al. (2020a), Lu et al. (2020)
NRF2, PPARG.	Activation of the NLRP3 inflammasome, ROS, PCSK9	P2X7/LPS/NLRP3-PPARG	Inhibits oxidative stress in liver tissue	Yan et al. (2020)
SLC7A11/GSH/GPX4			Inhibits ferroptosis, significantly improves bone loss induced by NAFLD	Gu et al. (2024)
	ALT, AST, TC, LDL-C, TNF-α, IL-6, IL-1β, TLR4, MyD88 and NF-κB	TLR4/MyD88/NF-κB	Mitigates the progression and liver damage of NAFLD	Wang et al. (2020b)
AMPK	CCL19	TLR4/NF-κB-p65	Improves NAFLD	Zhao et al. (2018)
PRDM16, α-ketoglutarate, BAT		AMPK-PRDM16	Increases BAT quality and activity in mildly overweight NAFLD patients	Wu et al. (2019)
SIRT3, β-oxidation of fatty acids	LCAD		Attenuating high-fat diet-induced NAFLD in mice	Xu et al. (2019)
	Cols, MMP, Tgfβ1, Tgfbrs, Ctgf, α2, α-Sma, Loxl2, Long non-coding RNA H19, Tmsb10		Reduces immune cell infiltration, inhibits activation of neutrophils and expression of inflammatory genes, significantly suppresses inflammation, inhibits progression of NASH and hepatic fibrosis	Wang et al. (2021f)
Restores the Treg/Th17 ratio	Chemerin, CMKLR1, CCR2	Chemerin/CMKLR1	Reduces liver inflammation and lipid deposition, improves NASH	Lu et al. (2021)
	ROS	NOX2/FFA/ROS	Reduces the risk of progression to NASH and even cirrhosis	Sun et al. (2017b)
GSH, SOD, CAT	NO,TGF-β1, TNF-α		Reduces severe lipid peroxidation induced by PCM and prevents hepatocyte damage	Shams et al. (2024)
CYP7A1	SCP2, cholesterol transport to the plasma membrane	AKT/mTOR	Mitigates prostaglandin synthesis mediated by COX2, improves hepatic autophagy flux, thus regulating cholesterol homeostasis	Sun et al. (2018b)
*Actinomyces*, Romboutsia, ASBT, FGF19, CYP27a1, ABCB11 mRNA, Bacteroides-salanitronis-DSM-18170	Hydroxycinnamic acid, dehydrononalkaline, leucine, Desulfovibrio-piger	FAS, SREBP-1c, FXR, CYP27a1	Prevents fatty liver hemorrhagic syndrome by regulating gut microbiota and bile acid metabolism	Cheng et al. (2024), Wang et al. (2023a)

### 7.5 Protective effects on the reproductive system

**FIGURE 6:**
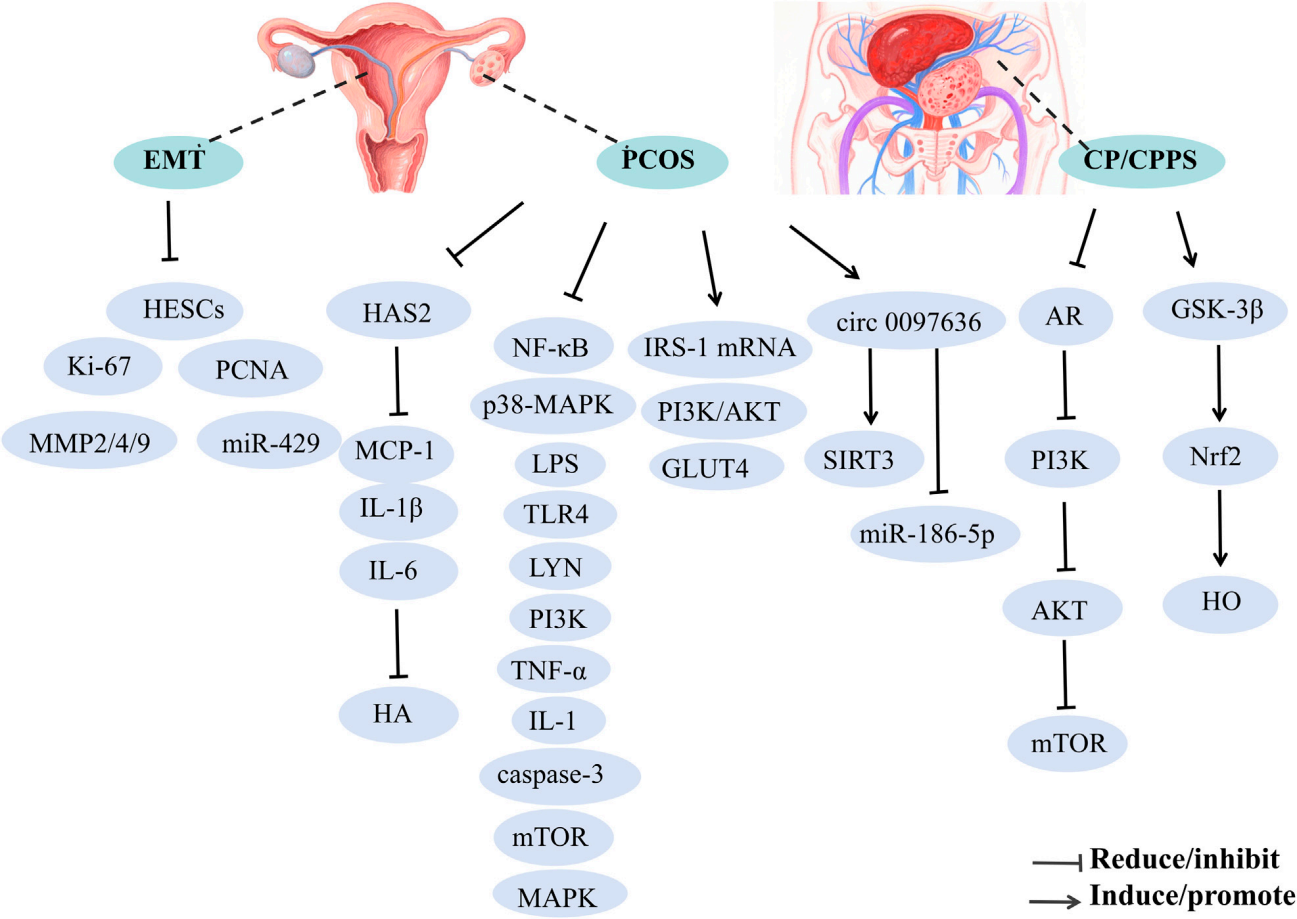
The mechanism of berberine’s action on the Reproductive system.

Reproductive system diseases affect the function and structure of reproductive organs, including conditions like male prostatitis, orchitis, female vaginitis, and uterine fibroids. These conditions can lead to pain, infertility, and menstrual disorders, severely affecting patient quality of life and reproductive health. This section briefly discusses the treatment of polycystic ovary syndrome (PCOS) (Jin et al., 2024), endometriosis (EMT) (Gu and Zhou, 2021), and chronic prostatitis/chronic pelvic pain syndrome (CP/CPPS) (Tian et al., 2024) with BBR (Figure 6).

#### 7.5.1 Polycystic ovary syndrome

BBR has been shown to ameliorate symptoms associated with PCOS, such as oligo-ovulation, embryo damage, and hormonal dysregulation, thereby contributing to its therapeutic effects. The underlying mechanisms may include: (1) upregulation of LHCGR, CYP19A1 (Wang Z. et al., 2021), and circ 0097636/SIRT3 (Wang S. et al., 2024), alongside downregulation of HAS2/MCP-1-IL-1β-IL-6 (He et al., 2024), integrin αvβ3, LPAR3, miR-186-5p, which further enhance endometrial receptivity, reduce hyaluronic acid (HA) synthesis, restore ovarian morphology, and improve DHT-induced KGN cell damage; (2) BBR alleviates cell apoptosis via ROS/caspase-3-dependent pathways, while inhibiting the NF-κB signaling pathway to reduce pro-inflammatory cytokines and mitigate LPS-induced embryo damage during pre-implantation stages (Miao and Cui, 2022); (3) BBR modulates hormonal imbalance and insulin resistance in PCOS rats by downregulating p38-MAPK and NF-κB proteins in ovarian tissues and lowering serum LPS levels (Zhao F. et al., 2022); (4) BBR exerts beneficial effects on PCOS through activation of the PI3K/AKT pathway, including modification of serum hormone levels, restoration of ovarian morphological changes, improvement of insulin resistance, and enhancement of cell viability while inhibiting apoptosis (Yu J. et al., 2021); (5) BBR potentially alleviates PCOS pathology and insulin resistance (IR) by suppressing apoptosis and modulating the expression of TLR4, LYN, PI3K, AKT, NF-κB, TNF-α, IL-1, IL-6, and caspase-3 (Shen HR. et al., 2021); (6) BBR enhances insulin sensitivity by increasing IRS-1 mRNA expression and decreasing mTOR mRNA levels, thereby improving therapeutic outcomes for PCOS (Kuang et al., 2020); (7) BBR may also mitigate PCOS by intervening in gut microbiota changes (Xin et al., 2024).

#### 7.5.2 Endometriosis

BBR significantly inhibits the proliferation and colony formation of human endometrial stromal cells (HESCs) by reducing the expression of proliferative markers, including Ki-67 and PCNA, as well as matrix metalloproteinases (MMP2, MMP4, and MMP9). This effect is mediated through the downregulation of miR-429, suppressing HESCs' proliferation, invasion, and migration (Gu and Zhou, 2021).

#### 7.5.3 Chronic prostatitis/chronic pelvic pain syndrome

BBH may alleviate symptoms of CP/CPPS by modulating gut microbiome signaling. Notably, butyrate-producing bacteria have been identified as key players, mediating the alleviation of CP/CPPS through the inhibition of the AR-PI3K-AKT-mTOR pathway and the activation of the GSK-3β-DUSP1-Nrf2-HO axis. Additionally, BBH mitigates prostatitis, suppresses lipid peroxidation and oxidative stress, reduces pro-inflammatory cytokines, and significantly lowers the prostate index in CP/CPPS rats (Tian et al., 2024).

### 7.6 Antitumor activity

BBR exhibits extensive antitumor properties primarily through mechanisms that induce apoptosis and autophagy, inhibit tumor proliferation, metastasis, and invasion. The mechanisms include: (1) induction of apoptosis and autophagy via NRF2 degradation (Guan et al., 2020), activation of ATF6/GRP78 (La et al., 2017), and downregulation of the AKT/mTOR/GLUT1 signaling pathway (Guo et al., 2021), which reverses the Warburg effect; (2) inhibition of proliferation and metastasis by suppressing Topoisomerase I, DSBs, and MUS81-EME1 (Inoue et al., 2021), downregulating KRAS expression, and impeding KRAS-G4 replication, thereby delaying DNA synthesis (Wang KB. et al., 2022), disruption of claudin interactions and promotion of immune macrophage transformation (Cornelius et al., 2022; Shah et al., 2022); (3) modulation of molecular interactions by interacting with microRNAs to suppress cell proliferation and telomerase activity, and activating AMPK while inhibiting ACC to reduce fatty acid synthesis and vesicular secretion (Gu et al., 2020); (4) alleviation of oxidative stress by promoting Dicer expression, reducing DNA damage, and mitigating inflammation to suppress carcinogenesis (Wu et al., 2020); (5) reversal of exosome function by inhibiting the tumorigenic effects of colon cancer exosomes, reducing cell survival and metastasis in colorectal cancer (CRC) (Sun Q. et al., 2023).

**FIGURE 7:**
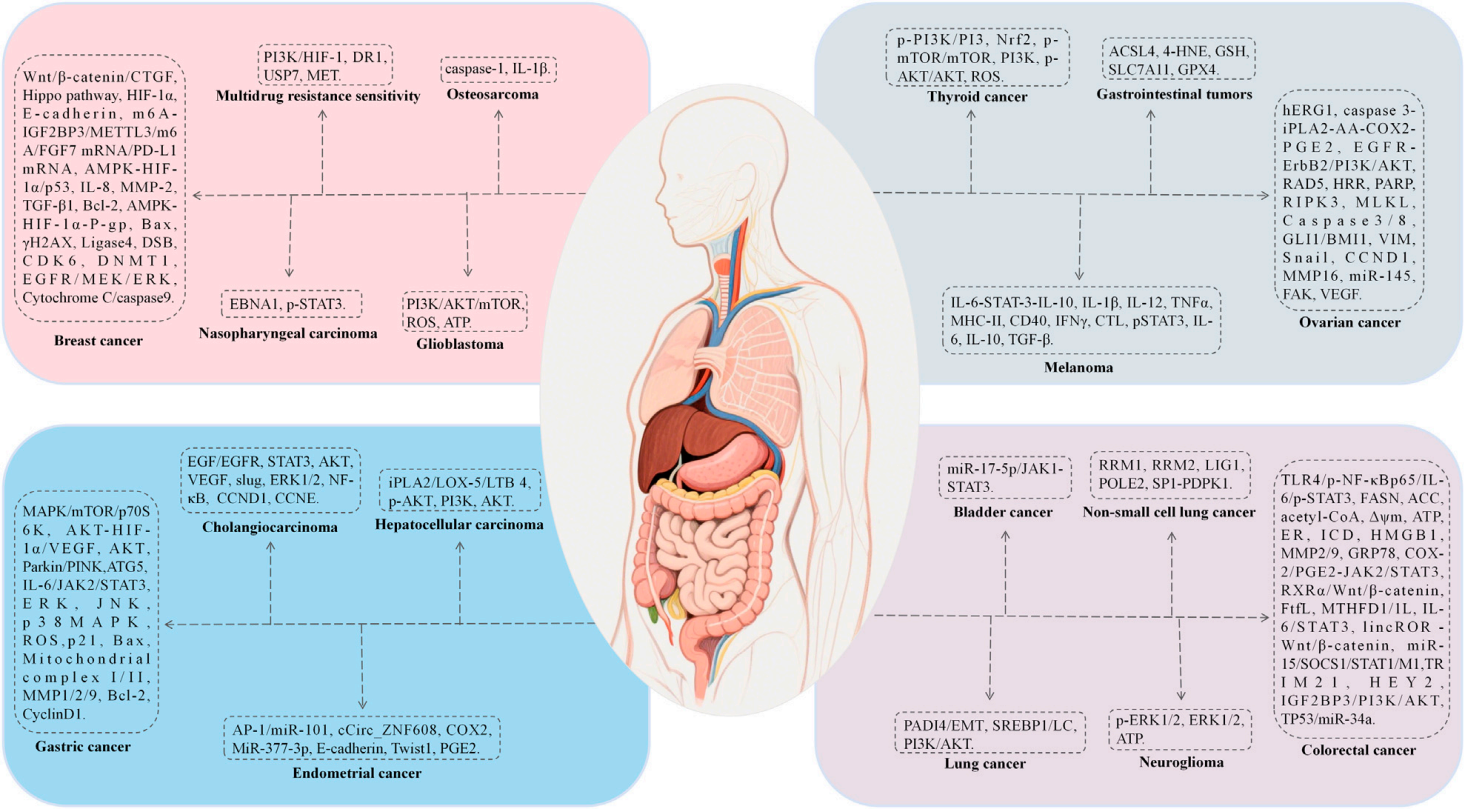
The mechanism of berberine’s action on cancer.

We have summarized the pharmacological mechanisms of BBR against various tumors, including rhabdomyosarcoma, melanoma, glioma, lung cancer, breast cancer, hepatocellular carcinoma, and colorectal cancer (Figure 7; Table 9).

**TABLE 9:** The mechanism of berberine’s action on cancer.

Disease	Promote/increase	Suppression/reduction	Conditioning pathway	Implication	References
Melanoma	IL-1β, IL-12, TNFα, MHC-II, CD40, IFNγ, CTL.	pSTAT3, IL-6, IL-10, TGF-β	IL-6-STAT-3-IL-10	BBR restores Tcell anti-tumor cytotoxicity in the tumor microenvironment	Shah et al. (2022)
Osteosarcoma		caspase-1, IL-1β		Inhibits the growth of tumor cells	Jin et al. (2017)
Glioblastoma	ROS	Mitochondrial count, ATP	PI3K/AKT/mTOR	Induces mitochondrial dysfunction	Maiti et al. (2019), Palma et al. (2020)
Neuroglioma		p-ERK1/2, ATP, Mitochondrial aerobic respiration, ERK1/2		Inhibits mitochondrial aerobic respiration, reduces ATP production	Sun et al. (2018c)
Gastrointestinal tumors	ACSL4, 4-HNE	GSH, SLC7A11, GPX4		Disrupts the antioxidant mechanisms of tumor cells	Mori et al. (2023)
Lung cancer	CD86	PADI4/EMT, PADI4/I RF5, CD163, CD206		Reverses macrophage functions associated with PADI4	Gu et al. (2022), Liao et al. (2024), Wang et al. (2023b)
	ROS, H2O2	SREBP1/LC		Inhibits cell proliferation in A549 and H1299 cells	Liao et al. (2024)
		KIF20A, CCNE2	PI3K/AKT	Induces apoptosis	Wang et al. (2023b)
Non-small cell lung cancer		RRM1, RRM2, LIG1, POLE2, SP1-PDPK1		Reduces cell growth, migration, and invasion	Ni et al. (2022a), Zheng et al. (2018)
Hepatocellular carcinoma		p-AKT, PI3K	AKT	Inhibit cell growth, migration, and invasion	Song et al. (2019)
		iPLA, LOX-5, LTB4	iPLA2/LOX-5/LTB4	Reverses adhesion and migration of HepG2 cells	Zhao et al. (2020)
Gastric cancer		AKT, HIF-1α, VEGF	AKT-HIF-1α/VEGF	Reverses gastric mucosal atrophy	Ye et al. (2024)
	p21, Bax	IL-6, Phosphorylation of JAK2, STAT3, Bcl-2, CyclinD1	IL-6/JAK2/STAT3	Inhibits proliferation, migration, and invasion of gastric cancer cells	Xu et al. (2022), Zhang et al. (2020), Yang et al. (2018)
	mTOR, p70S6K	AKT, ERK, JNK, p38MAPK	MAPK/mTOR/p70S6K	Inhibits autophagy	Zhang et al. (2020)
	Mitochondrial complex II, Parkin/PINK, ATG5, ROS	Mitochondrial complex I, MMP1, 2, 9		Damages the antioxidant system, reduces mitochondrial membrane potential	Lin et al. (2008), Warowicka et al. (2019)
Colorectal cancer		TLR4, p-NF-κB, p65, IL-6, p-STAT3	TLR4/p-NF-κBp65/IL-6/p-STAT3	Inhibits proliferation of colorectal tissue cells	Yan et al. (2022)
		FASN, ACC, acetyl-CoA		Reduces lipids to inhibit proliferation of cells	Li et al. (2023b)
	Continuous release of Ga^2+^ from the endoplasmic reticulum, ER, ICD, Number of phagocytic cells, HMGB1	Caspase activity, Δψm, ATP		CRT surface exposure and translocation, induces oxidative stress, programmed cell death	Mianowska et al. (2023)
		COX-2/PGE2, Phosphorylation of JAK2 and STAT3, MMP2/9, GRP78	COX-2/PGE2-JAK2/STAT3	Inhibits *in vitro* and *in vivo* growth/migration/invasion of CRC cells	Liu et al. (2015)
	c-Cbl	β-catenin	RXRα/Wnt/β-catenin	Induces degradation of β-catenin proteasome	Ruan et al. (2017)
		FtfL, Nucleocytovirus, symbiotic *Lactobacillus*, lactic acid *Lactobacillus*, *Vibrio*, MTHFD1/1L		Weakens the B-cell-mediated immune modulation of CRC induced by *Vibrio*	Yan et al. (2023), Qian et al. (2023)
		IL-17, LPC	IL-6/STAT3, lincROR -Wnt/β-catenin	Repairs intestinal barrier function	Chen et al. (2023)
	SOCS1	miR-15, STAT1	miR-15/SOCS1/STAT1/M1	Inhibits M1 polarization of macrophages	Ling et al. (2023)
	SUFU	Bcl-2, Bax, MMP, SHH, Ptch1, SMO, Gli1, c-Myc, cyclin D1	Hedgehog signaling cascade	Induces mitochondrial-mediated apoptosis in CRC cells	Shen et al. (2021b)
	TRIM21 degradation of IGF2BP3	Stabilizing effect of IGF2BP3 on CDK4/CCND1 mRNA.	Ubiquitin-proteasome, IGF2BP3/PI3K/AKT	Inhibits proliferation and induces G1/S phase arrest in CRC cells	Gui et al. (2023)
	Arntl, Clock, Nr1d1, Wnt, ISC.	Activation of macrophages and granulocytes		Promotes regeneration of colitis epithelium	Luo et al. (2022)
		HEY2, E-cadherin, β-catenin, Cyclin D1		Inhibits survival, invasion, and migration of CRC cells	Ni et al. (2022b)
	NF-κB, OCLUDIN, ZO-1	MMP9, Ereg, Muc16, IL-1b, TNF-α, EphA2, Sema7a, MMP13, Dusp10, Ki-67, COX-2, IL-β, p-JNK, p-STAT3, c-Myc	Gut microbiota-amino acid metabolism-Wnt signaling axis	Regulates gut microbiota to suppress pro-inflammatory genes and carcinogenic factors, thereby inhibiting CRC growth in conventional mice	Chen et al. (2022a), Deng et al. (2022), Nie et al. (2022), Chen et al. (2020b)
	lncRNA CASC 2	BCL2		Induces Bcl-2 translational inactivation mediated	Dai et al. (2019)
	STAT3, NF-κB, TP53, miR-34a	ALX, KRAS.	TP53/miR-34a	Enhances the antitumor activity of MIA-PaCa-2+ pLXSN cells	Akula et al. (2020), Rigillo et al. (2024)
Cholangiocarcinoma		EGF/EGFR, STAT3, AKT, VEGF, slug, ERK1/2, NF-κB, CCND1, CCNE.		Blocks G1 phase of cancer cells, inhibits growth, migration, and invasion of cholangiocarcinoma	Obchoei et al. (2022), Puthdee et al. (2017)
Thyroid cancer	ROS	p-PI3K/PI3, p-mTOR/mTOR, PI3K, p-AKT/AKT, Nrf2		Dose-dependently inhibit the proliferation of K1 thyroid cancer cells induced by high glucose	Shi et al. (2023), Ni et al. (2017)
Breast cancer	Hippo pathway, HIF-1α, E-cadherin, CTGF.	Wnt/β-catenin pathway	Wnt/β-catenin/CTGF.	Inhibits proliferation, migration of cancer cells	Sammarco et al. (2023), Sun et al. (2023b)
	FGF7 mRNA	PD-L1 mRNA	m6A-IGF2BP3/METTL3/m6A/FGF7 mRNA/PD-L1 mRNA.	Halts the progression of breast cancer	Fu et al. (2024)
	p53	AMPK, HIF-1α	AMPK-HIF-1α/p53, AMPK-HIF-1α-P-gp	Reverses hypoxia-induced chemotherapy resistance in BC treated with doxorubicin	Pan et al. (2017a), Pan et al. (2017b)
Triple-negative breast cancer	Fibroblast growth factor, angiogenesis biomarker genes	CDK6, DNMT1		Reduces cell proliferation	Jabbarzadeh et al. (2022)
	Induces double-strand breaks, release of cytochrome C, caspase-3/9, Bax, γH2AX, Ligase4, DSB	EGFR, IL-8, MMP-2, TGF-β1, Bcl-2, MEK and ERK phosphorylation	Cytochrome C/caspase 9, EGFR/MEK/ERK.	Inhibits cell invasion and growth of TNBC	Kim et al. (2018a), Zhao et al. (2017b), Kim et al. (2018b)
Endometrial cancer	MiR-377-3p, E-cadherin	cCirc_ZNF608, COX2, Twist1, PGE2	AP-1/miR-101	Inhibits the growth, invasion, and metastasis of EC	Liang et al. (2022)
Ovarian cancer	miR-145	MMP16, EGFR, ErbB2, CCND1, MMP, VEGF.	EGFR-ErbB2/PI3K/AKT	Inhibits proliferation, migration, and metastasis of cells	Li et al. (2021b), Chuang et al. (2021)
	RIPK3, MLKL, Caspase3/8	GLI1/BMI1, VIM, Snail, CCND1	hERG1	Induces G1 cell cycle arrest in EOC cells	Liu et al. (2019), Zhi et al. (2019)
		Phosphorylation of FAK, caspase 3, iPLA2, AA, COX2, PGE2	Caspase3-iPLA2-AA-COX2-PGE2	Inhibit the reproliferation of EOC cells induced by chemotherapy	Zhao et al. (2017c)
	PARP	RAD5, HRR		Induces oxidative stress and DNA damage	Hou et al. (2017)
Prostate cancer		Ribosomal protein S6K, Polθ, NF-κB/p62		Increases cancer cell sensitivity to radiation	Clark et al. (2024)
	Caspase-3	PSA, AR, COX-2, Bcl-2		Inhibit cell proliferation and induce cell apoptosis	Li et al. (2017)
Bladder cancer	miR-17-5p	JAK1, STAT3	miR-17-5p/JAK1-STAT3	Enhance the cytotoxicity of BC induced by gemcitabine	Li et al. (2023c), Xia et al. (2021)
Nasopharyngeal carcinoma		EBNA1, p-STAT3		Induces cell cycle arrest and apoptosis	Wang et al. (2017b)
Multidrug resistance sensitivity related to anticancer drugs		MDR1, USP7, MET		Overcomes osimertinib acquired resistance caused by MET amplification	Sun et al. (2024b), Chen et al. (2022b)
			PI3K/HIF-1	Overcomes radioresistance induced by low glucose and hypoxia	Zeng et al. (2020)

### 7.7 Antimicrobial activity

**FIGURE 8:**
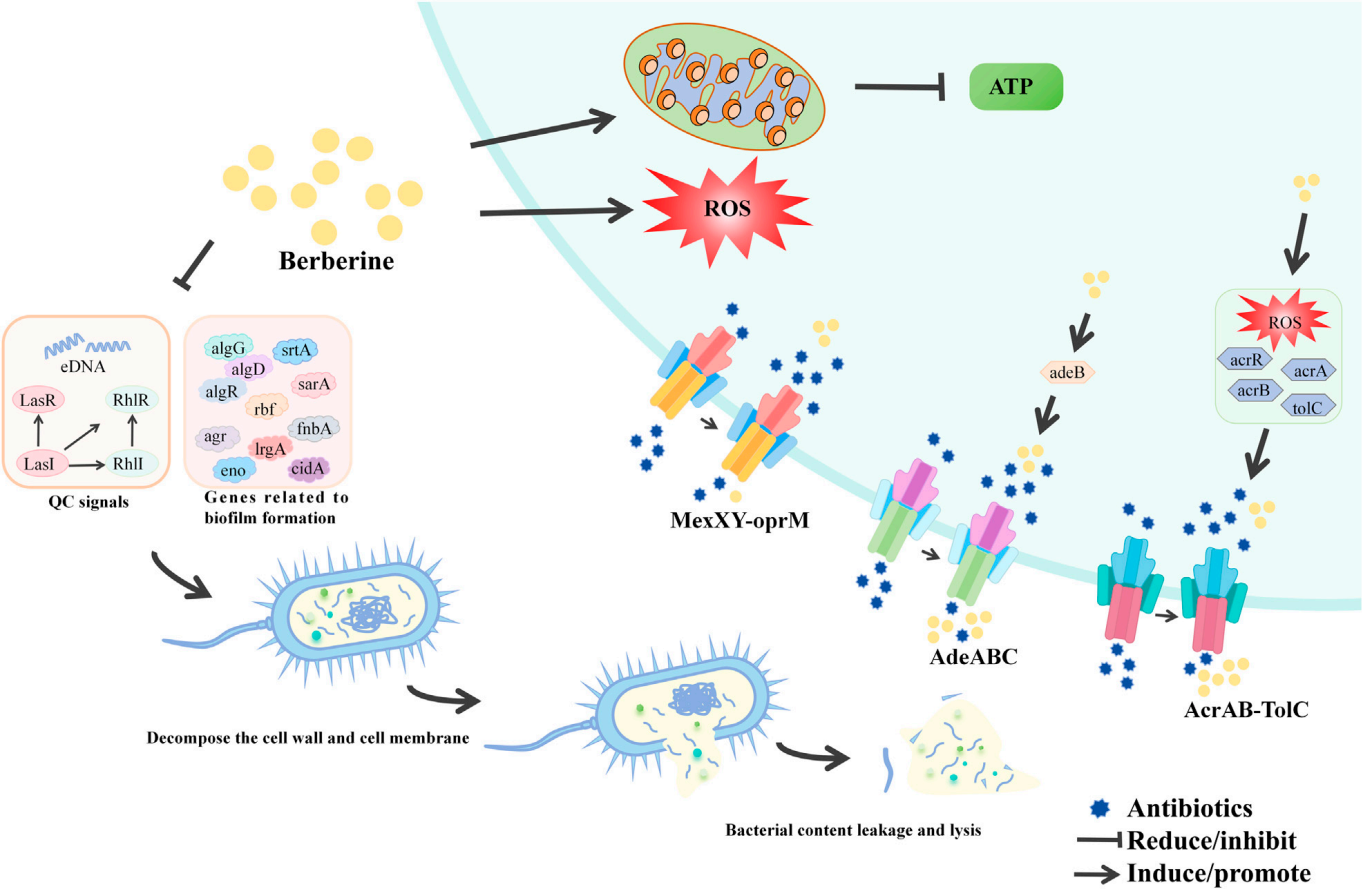
The antibacterial mechanism of berberine.

BBR exerts its antibiotic adjuvant effects through two main mechanisms. Firstly, it reduces the development of antibiotic resistance by inhibiting bacterial efflux pumps and biofilm formation. Secondly, BBR enhances antibiotic efficacy by interacting with host defense mechanisms and restoring the intestinal microbiota. The table below provides a brief overview of BBR’s antimicrobial activity according to bacterial species (Figure 8; Table 10).

**TABLE 10:** The antibacterial mechanism of berberine.

Microbial strains	Promote/increase	Suppression/reduction	Implication	References
*Pseudomonas aeruginosa*	Genes related to the AcrAB-TolC efflux pump: acrA, acrB, tolC, and acrR, ROS	ATP, PslA, PelA, Quorum sensing molecular level, lasI, lasR, rhlI, rhlR, eDNA, algG, algD, algR	Disrupts bacterial cell membranes, inhibits biofilm formation and maturation, enhances sensitivity to ciprofloxacin	Liu et al. (2024b), Aswathanarayan and Vittal (2018), Zhou et al. (2016b)
Methicillin-resistant *Staphylococcus aureus*	Disruption and dissolution of cell wall structure		Causes bacterial lysis and alters membrane permeability	Zhou et al. (2023)
*Staphylococcus aureus*		SrtA, agr, sarA, fnbA, rbf, lrgA, cidA, eno	Enhance its drug resistance	Ning et al. (2022)
*Salmonella Typhi*		Expression and number of type I fimbriae	Affects fimA gene expression, reducing the activity and adhesion of *Salmonella Typhi*	Xu et al. (2021b)
*Haemophilus* parasuis		PK-15	Affects outer membrane proteins, transferrins, and energy metabolism	Jia et al. (2021)
Fluconazole-resistant *Candida* albicans strains	Levels of DNA strand breaks		Induces loss of cell viability, plasma membrane damage, and mitochondrial dysfunction in *Candida* albicans	Da et al. (2016)
Oropharyngeal candidiasis	gC1qR-EGFR/ERK/c-Fos		Mediates endocytosis by oral epithelial cells in response to *Candida* albicans infection	Bao et al. (2024)
Antibiotic multidrug resistance pumps	AdeABC efflux pump gene adeB		Reduces the extrusion of antibiotics by the AdeABC pump	Li et al. (2021c)

### 7.8 Anti-inflammatory and antioxidant activity

BBR demonstrates multiple pharmacological actions, including the enhancement of antioxidant enzyme activity, direct scavenging of free radicals, and anti-inflammatory effects, all of which contribute to its potential therapeutic applications. The specific mechanisms underlying its antioxidant and anti-inflammatory effects may include: (1) the activation of endogenous antioxidant enzymes (SOD, CAT, GPx), the stimulation of the AMPK, PI3K/AKT, and Nrf2 pathways, alleviation of oxidative stress, inhibition of NADPH oxidase, and reduction of ROS levels, thus preventing oxidative damage (Carrizzo et al., 2020); (2) direct scavenging of free radicals by donating electrons or hydrogen atoms, forming coordination bonds with metal ions via hydroxyl and methoxy groups, and effectively sequestering metals such as iron and copper, thereby amplifying BBR’s antioxidant activity (García-Muñoz et al., 2024); (3) activation of the antioxidant Keap1/Nrf2/HO-1 pathway, which alleviates cholesterol overload-induced oxidative stress and apoptotic cell death in mouse hepatocytes, suggesting that BBR may be a potential therapeutic agent for cholesterol-related cardiovascular diseases (Ye et al., 2022); (4) inhibition of pro-inflammatory cytokines (e.g., IL-1β, IL-6, TNF-α), thereby reducing inflammation; suppression of NF-κB pathway activation, IKBα degradation, and MAPK pathway activation, while enhancing the STAT1 signaling pathway; BBR influences cellular physiological activities by directly interacting with the cell membrane through various mechanisms (Wang K. et al., 2024); (5) targeting IRGM1 to suppress the PI3K/AKT/mTOR pathway (Meng et al., 2024), as well as inhibiting IL-17 secretion and expression through the IL-17 signaling pathway, exerting anti-inflammatory effects. This study also indicates that BBR reduces the expression of CD^3+^, CD^4+^, CD^8+^, and Th^17+^ lymphocytes, as well as the expression of inflammatory cytokines IL-6, IL-8, and IL-17, contributing to its anti-inflammatory effect in the treatment of periodontitis (Li et al., 2024).

### 7.9 Other pharmacological mechanisms and disease treatments

#### 7.9.1 Acute lymphoblastic leukemia

BBR induces autophagic cell death in acute lymphoblastic leukemia (ALL) cells through inactivation of the AKT/mTORC1 signaling pathway (Liu et al., 2020). Additionally, BBR significantly increases the expression of caspases (CASP3, CASP8, CASP9) and pro-apoptotic genes (BAX, BAK1, BIK), while downregulating anti-apoptotic genes (BCL2, BCL2L2, BNIP1, BNIP3), thereby inducing apoptosis in leukemia cells via intrinsic pathways (Okubo et al., 2017).

#### 7.9.2 Acute graft-versus-host disease

BBR alleviates acute graft-versus-host disease (GVHD) by suppressing inflammation, remodeling the gut microbiota, and protecting the intestinal mucosal barrier. The specific mechanisms include: (1) reduction of GVHD-induced weight loss and GVHD index scores through the NF-κB pathway, alleviation of liver and intestinal damage, suppression of ALT and AST activity in the liver and intestines, and reduction of inflammation, oxidative stress, and NF-κB activation; (2) inhibition of inflammation and reduction of Th1 cell count, with downregulation of Th1 activation and alleviation of chronic GVHD (Wang M. et al., 2020); (3) remodeling the gut microbiota and protecting the intestinal mucosal barrier, significantly suppressing the activation of the NLRP3 inflammasome and the expression of inflammatory cytokines (such as IL-1β, IL-18, IFN-γ, TNF-α, MCP-1, and IL-6) (Zhao Y. et al., 2022), thereby protecting against GVHD through inhibition of inflammasome activation by “Signal 1” and “Signal 2.”

#### 7.9.3 Kawasaki Disease

BBR protects patients with Kawasaki Disease (KD) by mitigating inflammatory responses, inhibiting oxidative stress and ER stress, and reversing endothelial progenitor cell (EPC) proliferation. The mechanisms include: (1) accelerating the reduction of CRP, NLR, and PLR levels, thus alleviating inflammation (Fan et al., 2021); (2) reducing ROS production and the expression of ER stress-related proteins (e.g., ATF4, p-EIF2α, p-PERK, XBP1, p-IRE1, HSP90B1, HSPG2, DNAJC3, P4HB, and VCP), protecting KD-induced human coronary artery endothelial cells (HCAECs) from apoptosis and regulating the cell cycle, arresting cells in the G0/G1 phase; (3) BBR reverses impaired EPC proliferation during the acute phase of KD, by activating the PI3K/AKT/eNOS signaling pathway and increasing PI3K/AKT/eNOS mRNA levels, as well as protein levels of PI3K, p-AKT, eNOS, and p-eNOS (Xu et al., 2020; Xiao et al., 2014).

#### 7.9.4 Ferroptosis

BBR inhibits ferroptosis by modulating Nrf2 transcription, activating the Nrf2 signaling pathway, and increasing the expression of GPX4, FPN1, and SLC7A11 (NRF2/SLC7A11/GPX4 pathway). This reduces levels of iron, MDA, and ROS, stabilizing atherosclerotic plaques (Li X. et al., 2023). Moreover, BBR suppresses the interaction between Keap1 and Nrf2, partially preventing RSL3-induced ferroptosis through activation of Nrf2 signaling. Additionally, BBR alleviates neuronal ferroptosis in spinal cord injury rats via the AMPK-NRF2-HO-1 pathway, and regulates the Circ_0097636/MiR-186-5p/SIRT3 pathway to prevent dihydrotestosterone-induced granulosa cell damage and ferroptosis (Song et al., 2023).

## 8 Critical assessment

The research on BBR has advanced from the stage of “activity discovery” to the “deep waters” of clinical translation. However, its clinical transformative value is constrained by systemic methodological flaws, despite its biological activity being investigated across multiple systemic diseases.

The current clinical translation of BBR faces multi-dimensional and systematic limitations. At the model level, existing animal models are mostly simplified simulations of human diseases, making it difficult to reproduce the multi-dimensional characteristics of diseases, such as the superimposition of age-related pathologies and the dynamic interaction between the immune system, microbiota, and host. This leads to significant “model dependence” in mechanism conclusions, which are disconnected from clinical phenotypes. In terms of dose-effect, the oral bioavailability of BBR is extremely low (<1%), resulting in a contradiction between *in vitro* effectiveness and *in vivo* ineffectiveness. Moreover, high doses pose an oxidative stress risk, and the dose-effect relationship lacks a systematic assessment based on pharmacokinetics, which restricts the selection of clinical doses.

There are obvious hierarchical deficiencies in clinical evidence. Existing randomized controlled trials are mostly small-sample and short-term designs, making it difficult to support the long-term application value of BBR in chronic diseases. At the same time, there are issues such as racial bias and insufficient control of confounding factors, which weaken the universality of the conclusions. Additionally, there is insufficient research on the interaction between BBR and conventional drugs, affecting clinical guidance for combined medication. Mechanism research has long focused on classic pathways such as NF-κB and AMPK, lacking systematic integration across pathways and organs. The verification of causal relationships in the interaction between microbiota and host is also insufficient.

To overcome the aforementioned bottlenecks, the implementation of a “three-pronged breakthrough” strategy emphasizing multidisciplinary collaboration is essential. In terms of model innovation, efforts should focus on developing humanized organoids, immunocompetent tumor models, and multi-factor-induced composite models to reduce reliance on traditional models and better reflect human pathological features. Optimization of delivery systems should leverage nano-targeting technologies (Hsu et al., 2024), liposomes (Mianowska et al., 2023), solid dispersion techniques (Leimann et al., 2023), enteric-coated pellets, or prodrug strategies to enhance lesion-specific accumulation. These approaches should be integrated with pharmacokinetic-pharmacodynamic modeling to more accurately define the effective dose range. In clinical research, there is a need to transition toward multicenter, large-sample, long-term follow-up randomized controlled trials (RCTs) that prioritize hard clinical endpoints. These trials should incorporate biomarker or microbiota profiling for patient stratification and enable a systematic evaluation of the safety profile of combination therapies.

## 9 Conclusion

This review’s emphasis on the “pharmacological effects” of BBR might have resulted in an insufficiently in depth exploration of its “hurdles in clinical applications”.

Specifically, problems like BBR’s low bioavailability and formulation constraints (for example, the formulation challenges arising from its poor solubility) were merely touched upon in the discussion. However, they were not systematically analyzed in light of current research efforts, such as the exploration of delivery systems including nanoformulations and prodrug modifications. As a consequence, it is arduous to comprehensively represent the bottlenecks in its translation from the laboratory to the clinical setting. Moreover, there is a dearth of comparative research on BBR and other natural products (such as berberine analogues), which precludes the clear demonstration of its distinctive advantages and limitations.

In conclusion, future research endeavors should prioritize overcoming the bottlenecks in clinical translation, such as optimizing the route of administration and conducting multi center RCTs. Additionally, efforts are needed to elucidate the core mechanisms of action, for example, by validating key targets through gene knockout or knock in techniques. Furthermore, it is essential to intensify research on long term safety and the applicability in special populations. These measures are crucial for facilitating BBR’s transition from a “focal point in basic research” to a “clinically efficacious drug.”
